# Deprivation and limitations in daily life in new onset kidney disease: a population study

**DOI:** 10.1093/ckj/sfaf397

**Published:** 2025-12-17

**Authors:** Eilidh Cowan, Samira Bell, Corri Black, Tom Blakeman, Simon Fraser, Audrey Hughes, Buse Keskindag, Shona Methven, Mintu Nath, Dorothea Nitsch, Magdalena Rzewuska Diaz, Nicole Scholes-Robertson, Simon Sawhney

**Affiliations:** Aberdeen Centre for Health Data Science, University of Aberdeen, Aberdeen, UK; Division of Population Health and Genomics, School of Medicine, University of Dundee, Dundee, UK; Renal Unit, Aberdeen Royal Infirmary, NHS Grampian, Aberdeen, UK; Centre for Primary Care, Institute of Population Health, University of Manchester, Manchester, UK; School of Primary Care, Population Sciences and Medical Education, University of Southampton, Southampton, UK; Grampian Kidney Patient Association, Aberdeen, UK; Aberdeen Centre for Evaluation, University of Aberdeen, Aberdeen, UK; Renal Unit, Aberdeen Royal Infirmary, NHS Grampian, Aberdeen, UK; Aberdeen Centre for Health Data Science, University of Aberdeen, Aberdeen, UK; UK Kidney Association, Bristol, UK; Faculty of Epidemiology and Population Health, London School of Hygiene and Tropical Medicine, London, UK; Aberdeen Centre for Evaluation, University of Aberdeen, Aberdeen, UK; Sydney School of Public Health, University of Sydney, Sydney, Australia; Aberdeen Centre for Health Data Science, University of Aberdeen, Aberdeen, UK; Renal Unit, Aberdeen Royal Infirmary, NHS Grampian, Aberdeen, UK

**Keywords:** chronic kidney disease, deprivation, equity, health inequalities, social determinants

## Abstract

**Background:**

Existing population research has evaluated inequities in health outcomes for people in deprived communities who have early kidney disease, but not the differences in their self-reported overall health and ability to manage daily life activities when they first present, or the additional burden for people of working age. Using their responses to the national Census in Scotland, we studied the self-reported overall health and impact on day-to-day life of people in deprived and affluent households who newly presented with evidence of kidney disease.

**Methods:**

Of 458 897 adult North Scotland residents, we included all 24 775 individuals who presented with new onset kidney disease (eGFR <60 ml/min/1.72 m^2^) in 2011–2014. We measured deprivation based on household (Census) and resident neighbourhood (index of multiple deprivation). We fitted proportional odds regression models that accounted for age, sex, comorbidities, and additional impairments (e.g. vision, hearing, learning difficulties). We further adjusted for self-reported mental health and living alone as potential mediators, and tested for interactions with working age (18–65 years), sex, and mental health.

**Results:**

Of 24 775 people newly presenting with kidney disease, already 11 115 (45%) reported limitations in their daily lives. People in the most deprived (vs least) neighbourhoods and households experienced 2-fold greater odds of worse self-reported health (adjusted odds ratio, OR 2.05, 1.81–2.32 neighbourhood; OR 1.93, 1.64–2.26 household); and greater limitation in day-to-day activities (OR 1.70, 1.49–1.95 neighbourhood; OR 1.65, 1.39–1.96 household). This pattern of inequity was even more pronounced (3-fold) among those of working age (interaction *P *< .0001).

**Conclusion:**

The association of deprivation with health and daily life represents an additional dimension of health inequity that is substantial, and evident from the earliest stages for people with kidney disease.

## GRAPHICAL ABSTRACT

**GRAPHICAL ABSTRACT:**
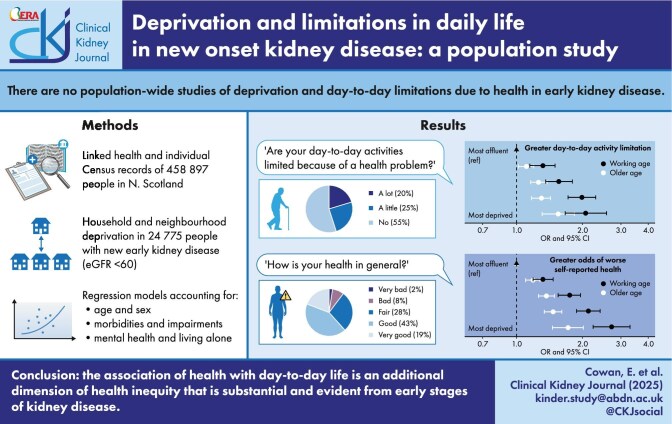

KEY LEARNING POINTS **What was known:**

- Previous studies show that people from deprived communities experience poorer health outcomes when they newly present with kidney disease.
- While we could find selective surveys of people already under nephrology care, we identified no general population studies exploring how deprivation in early kidney disease affects self-reported health and day-to-day life.

**This study adds:**

- We looked at how people with new kidney disease in Scotland rated their own health and ability to manage daily activities. To do this, we used responses to a national Census, which informs key UK policies on health and wellbeing.
- Even in these early stages of kidney disease, many people reported that their health was limiting their day-to-day living: nearly half of respondents said so.
- No matter how we measured deprivation, the pattern was clear: people living in deprived communities were twice as likely to report poor health and limitations in daily life. Among people of working age, this gap was even wider: they were three times as likely to be affected.

**Potential impact:**

- The association of deprivation with self-reported general health and day-to-day living represents an additional dimension of kidney health inequity that is underreported, substantial, and evident from the earliest stages of newly presenting with kidney disease.
- These are pressing inequities that warrant attention to ensure that we can organize our kidney services across primary, secondary, and social care so that they may holistically address the challenges faced by disadvantaged and under-served communities.

## INTRODUCTION

People with kidney disease typically first hear about their condition and receive initial care in primary care or from non-specialists. Clear framing in guidelines and patient information about what kidney disease means for everyday life is therefore crucial: both to support early recognition and to assist the often-complicated initial discussions about what to expect [1, 2]. Generally, patient-facing information about kidney disease describes that ‘there are usually no symptoms of kidney disease in the early stages’ [3, 4]. This may be the case for many people but may be problematic for those who do have symptoms if they consequently believe that their experiences are not relevant or legitimate to discuss. Extrapolation of existing research would suggest that people newly presenting with kidney disease may already experience symptoms, such as in the context of living with multiple long-term conditions (multimorbidity) [5–9], however, most existing research relies on surveys and interviews of recruited subsets of people at later stages of kidney disease followed-up by kidney specialists, and studies with a focus on deprivation and equity are particularly sparse [9].

Equity and person-centredness of care may be evaluated within a context of the physical, psychological, social, and economic challenges, needs, and disadvantages that people may face [10]. Even in high-income countries, kidney health outcomes are inequitable [11–23], but existing epidemiological studies of the equity/inequity of kidney health have focused predominantly on medical processes and outcomes (e.g. mortality) and not on daily life. Associations between deprivation and daily life have been reported for some health conditions (cancer and cardiovascular diseases), but aside from subsets of surveys of people already under nephrology care, we were unable to find any previous population-wide studies covering people with early-stage kidney diseases [24–27]. Despite the limited epidemiological research, international stakeholders recently agreed that one of four critical core outcomes important to people with chronic kidney disease (CKD) was ‘life participation’ (alongside kidney, cardiovascular, and mortality outcomes) [28].

If we frame kidney care and equity in isolation and only in medical terms, we may miss opportunities to advocate for people with kidney diseases and implement holistic models of care that support those in greatest need [29]. As kidney disease leading to reduced kidney function affects at least 5% of people and typically occurs in the context of multiple long-term conditions [30], a burden on daily life may be common, be present from the point of first presentation, and be inequitable. In this study we used linked population health and social data to understand (i) the overall burden of self-reported health and limitation of daily life for people who newly present with kidney disease; (ii) the extent to which it may be worse for people in deprived circumstances; and (iii) whether any association could be explained by case-mix differences including age, morbidity, and physical impairments; or by other living circumstances including mental health, or living alone.

## MATERIALS AND METHODS

### Study population: people with new presentations of kidney disease within GLOMMS-CORE

GLOMMS-CORE is a population study of linked kidney health data and Census records to study the health and living circumstances of all adults in the North of Scotland (Grampian). Scotland is a high-income country but has the worst life expectancy in Western Europe with widening inequalities [31]. Within Grampian the largest city, Aberdeen, has a life expectancy at the median of Scotland (77 years for males, 81 years for females), with neighbourhoods both of marked deprivation and of marked affluence [32].

A compulsory Census in 2011 was completed by 90% of residents (458 897 in Grampian) [33]. To construct a cohort of people with new onset kidney disease based on their blood tests (eGFR results) we linked the Census to population-wide laboratory data covering all inpatient and community settings, as previously described elsewhere and summarized briefly in Supplementary Fig. 1 [14]. All laboratory results are reported through a single laboratory service for all of Grampian, enabling us to have complete capture of blood tests. Within our criteria, we did not require a proteinuria test, coding, or chronicity criteria for CKD, because proteinuria is tested only in a minority of people with kidney disease in the UK. Similarly, repeat tests and coding are frequently not completed, which would have led to a selection bias affecting the under-served populations we were most interested in [30]. To mirror the point when a first discussion about kidney health might occur, we studied those with new (incident) evidence of reduced kidney function (eGFR <60 ml/min/1.73 m^2^) in 2011–2014 and their recent Census responses. This strategy of focusing on new presentations was determined *a priori* through consultation with patient and clinical expert stakeholders, and ensured that we did not overstate the burden of ill health at first presentation by mixing prevalent and incident patients (Supplementary Fig. 2). To further mitigate any possible imbalance between deprived and affluent groups, we also adjusted for year of presentation in our regression models. Separately we also provided the self-reported health of those crossing different incident thresholds (eGFR <45, <30, first development of acute kidney disease) for context.

### Exposures: household and neighbourhood socioeconomic deprivation

To measure household socioeconomic deprivation, we used the National Statistics Socio-Economic Classification based on the occupation of the household reference person at the time of the 2011 Census [34] (Supplementary Table S1).

For neighbourhood deprivation we reported quintiles of the Scottish Index of Multiple Deprivation (SIMD) [35]. This was determined from resident postcode at kidney presentation, and represents an aggregate measure given to a neighbourhood based on seven domains: income, employment, health, access to services, education, housing, and crime. Quintile 1 refers to the most deprived 20% of neighbourhoods in Scotland and quintile 5 the most affluent 20%.

### Dependent variable: Self-reported health and limitation of day-to-day activities

The mandatory Census is central to policy decisions in the UK across society, businesses, councils, health services and charities, reporting on the wellbeing and needs of society. The Census included two questions covering overall health and impact on daily living, respectively [33]:

(i) ‘How is your health in general?’ (‘Very bad; Bad; Fair; Good; Very good’)
(ii) ‘Are your day-to-day activities limited because of a health problem or disability which has lasted, or is expected to last, at least 12 months?’ (‘Yes, limited a lot; Yes, limited a little; No’)

For context, within the underlying general population 17.8% of people gave a response of ‘not good’ for question (i), and 19.6% reported some level of limitation of day-to-day activities for question (ii) [36].

### Covariables

We included age, sex, and comorbidities from routine medical records, in addition to year of presentation. Comorbidities in the analyses were previous history of cancer, chronic obstructive pulmonary disease, coronary heart disease, diabetes, heart failure, hypertension, liver disease, peripheral arterial disease, atrial fibrillation, and stroke based on hospital episode ICD-10 codes available back to 2004, as described in previous work [14]. In addition to comorbidities as recorded in health episodes, we also reported patient-reported morbidities and social circumstances as recorded in the Census: presence of a visual impairment, hearing impairment, learning difficulty or disability, difficulty with English language, access to a car, rurality based on home postcode, presence of a self-reported mental health condition, and if the person reported living alone [37, 38].

### Analyses

For the main analysis cohort (people with new eGFR <60 ml/min/1.73 m^2^), we described the characteristics, and deprivation categories of people by their self-reported health status and level of day-to-day activity limitation both as crude proportions and age-standardized. Direct age standardization was performed using the most affluent group (i.e. professionals or SIMD quintile, respectively) as the reference group. For additional context we also provided age-standardized self-reported health and activity limitation for people presenting at each kidney threshold (eGFR <60, <45, <30, AKD).

To assess the relationship of health and deprivation we used a proportional odds regression model, using the MASS package in R [39]. Proportional odds regression is related to logistic regression but handles outcomes that have more than two ordered levels of severity [40, 41]. We used this approach to determine whether those living in areas or households of higher deprivation were more or less likely to report ‘worse’ self-reported health, and/or greater limitation of day-to-day activity than those from less deprived areas or households. We confirmed the inherent proportional odds assumption with a Brant–Wald test [42]. We evaluated for associations between neighbourhood/household deprivation, and self-reported health/activity limitation, with three levels of model adjustment that are outlined in a conceptual diagram (Supplementary Fig. 3): Stage A: age, sex, and presenting year; Stage B: comorbidities and physical impairments; and Stage C: mental health and social isolation (living alone) as potential mediators. *A priori*, we also incorporated tests for three separate interactions (dotted lines in Supplementary Fig. 3) using likelihood ratio tests of nested models to assess for different relationships according to female/male sex, mental health, and working/older age (18–65 vs 66–99 years). In advance, we determined that a likelihood ratio *P *< .1 would lead to stratification by sex, mental health, or age, respectively. All analyses were conducted in R version 4.4.1.

## RESULTS

### Population characteristics

**Figure 1:**
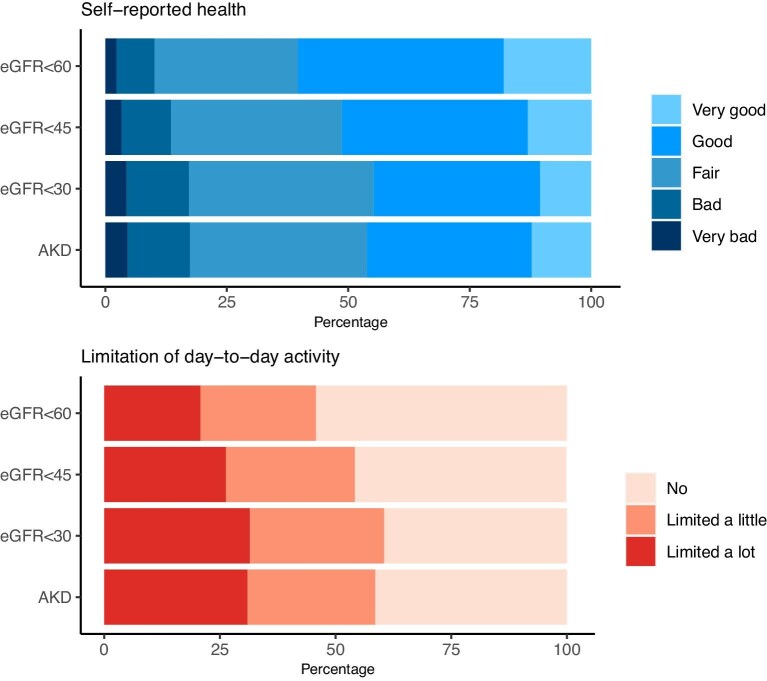
Age-standardized self-reported reported health and limitation of day-to-day activity among people presenting with kidney disease at different levels of severity (eGFR <60, <45, <30, and AKD).

Among 458 897 Census respondents, during 2011–2014 there were 24 775 new presentations (based on laboratory tests) of people with eGFR <60 ml/min/1.72 m^2^. The mean age was 70 years and 55% were female. Of respondents, 38% reported their health was not good, and 45% reported experiencing limitation in their day-to-day activities due to their health. While fewer people with early/mild threshold kidney disease (eGFR <60) reported reductions in health and limitation in daily life compared with those at more severe or acute kidney thresholds (eGFR <45, <30, <AKD), this difference was not substantial (Fig. 1). Worse self-reported health and activity limitation was reported by older people and those in deprived neighbourhoods and households, but did not differ by sex (Table 1). Those with comorbidities, including physical impairments and mental health conditions, or who lived alone also reported worse health and greater day-to-day limitation (Table 2).

**Table 1:** Population characteristics by self-reported health status and limitation of day-to-day activity.

		Self-reported health										Limitation of day-to-day activity					
	Total	Very bad		Bad		Fair		Good		Very good		Limited a lot		Limited a little		Not limited	
	*N*	N	Row %	*N*	Row %	*N*	Row %	*N*	Row %	*N*	Row %	*N*	Row %	*N*	Row %	*N*	Row %
Total	24 775	590	(2.4)	1960	(8.0)	6954	(28.0)	10 522	(42.5)	4749	(19.2)	5031	(20.3)	6124	(24.7)	13 620	(55.0)
Sex																	
Male	11 094	275	(2.5)	913	(8.2)	3131	(28.2)	4775	(43.0)	2000	(18.0)	2195	(19.8)	2652	(23.9)	6247	(56.3)
Female	13 681	315	(2.3)	1047	(7.7)	3823	(28.0)	5747	(42.0)	2749	(20.0)	2836	(20.7)	3472	(25.4)	7373	(53.9)
Mean age (SD)	70.9 (12.9)	68.9	(12.2)	69.9	(12.8)	73.5	(12.1)	71.4	(12.3)	66.8	(14.3)	73.9	(12.6)	74.1	(11.7)	68.4	(12.9)
Neighbourhood quintile (SIMD)																	
(most deprived) 1	1052	40	(3.8)	149	(14.2)	365	(34.7)	349	(33.2)	149	(14.2)	313	(29.8)	257	(24.4)	482	(45.8)
2	3839	131	(3.4)	418	(10.9)	1190	(31.0)	1508	(39.3)	592	(15.4)	945	(24.6)	965	(25.1)	1929	(50.2)
3	5279	146	(2.8)	452	(8.6)	1614	(30.6)	2170	(41.1)	897	(17.0)	1178	(22.3)	1334	(25.3)	2767	(52.4)
4	7219	164	(2.3)	510	(7.1)	1978	(27.4)	3119	(43.2)	1448	(20.1)	1338	(18.5)	1810	(25.1)	4071	(56.4)
(most affluent) 5	7386	109	(1.4)	431	(5.8)	1807	(24.5)	3376	(45.7)	1663	(22.5)	1257	(17.0)	1758	(23.8)	4371	(59.2)
Household NSSEC^a^																	
Professionals/Managerial	6515	123	(1.9)	384	(5.9)	1444	(22.2)	2906	(44.6)	1658	(25.4)	978	(15.0)	1521	(23.3)	4016	(61.6)
Intermediate/Small employer	5538	103	(1.9)	350	(6.3)	1413	(25.5)	2517	(45.4)	1155	(20.9)	951	(17.2)	1381	(24.9)	3206	(57.9)
Lower supervisory/technical	2933	84	(2.9)	263	(9.0)	907	(30.9)	1198	(40.8)	481	(16.4)	628	(21.4)	754	(25.7)	1551	(52.9)
Semi-skilled	4074	102	(2.6)	392	(9.6)	1195	(29.3)	1705	(41.9)	680	(16.7)	871	(21.4)	1036	(25.4)	2167	(53.2)
Unskilled	4579	142	(3.1)	448	(9.8)	1501	(32.8)	1815	(39.6)	673	(14.7)	1079	(23.6)	1131	(24.7)	2369	(51.7)
Never worked/unemployed	568	18	(3.2)	60	(10.6)	222	(39.1)	211	(37.1)	57	(10.0)	179	(31.5)	166	(29.2)	223	(39.3)

Abbreviation: NS-SEC, National Statistics Socioeconomic Classification.

a Total number is less than total as 568 individuals did not have assigned household classification.

**Table 2:** Comorbidities and social characteristics by self-reported health status and limitation of day-to-day activity.

		Self-reported health										Limitation of day-to-day activity					
	Total	Very bad		Bad		Fair		Good		Very good		Limited a lot		Limited a little		Not limited	
	*N*	*N*	Row %	*N*	Row %	*N*	Row %	*N*	Row %	*N*	Row %	*N*	Row %	*N*	Row %	*N*	Row %
Total	24 775	590	(2.4)	1960	(8.0)	6954	(28.0)	10 522	(42.5)	4749	(19.2)	5031	(20.3)	6124	(24.7)	13 620	(55.0)
Comorbidities at presentation																	
Diabetes	2344	116	(5.0)	372	(15.9)	900	(38.4)	817	(34.9)	139	(5.9)	780	(33.3)	668	(28.5)	896	(38.2)
COPD	2929	170	(5.8)	537	(18.3)	1105	(37.7)	897	(30.6)	220	(7.5)	1076	(36.7)	853	(29.1)	1000	(34.1)
Coronary heart disease	2312	96	(4.2)	292	(12.6)	871	(37.7)	830	(35.9)	223	(9.6)	699	(30.2)	704	(30.5)	909	(39.3)
Heart failure	1319	72	(5.5)	190	(14.4)	511	(38.7)	416	(31.5)	130	(9.9)	464	(35.2)	397	(30.1)	458	(34.7)
Hypertensive disease	6962	222	(3.2)	762	(10.9)	2465	(35.4)	2798	(40.2)	715	(10.3)	1884	(27.1)	2044	(29.4)	3034	(43.6)
Peripheral arterial disease	1102	64	(5.8)	188	(17.1)	413	(37.5)	346	(31.4)	91	(8.3)	426	(38.7)	344	(31.2)	332	(30.1)
Arterial fibrillation	2153	68	(3.2)	253	(11.8)	806	(37.4)	792	(36.8)	234	(10.9)	623	(28.9)	687	(31.9)	843	(39.2)
Stroke	1336	56	(4.2)	191	(14.3)	539	(40.3)	426	(31.9)	124	(9.3)	494	(37.0)	390	(29.2)	452	(33.8)
Cancer	3284	116	(3.5)	286	(8.7)	973	(29.6)	1343	(40.9)	566	(17.2)	641	(19.5)	845	(25.7)	1798	(54.7)
Mental health diagnosis																	
Yes	1231	100	(8.1)	300	(24.4)	527	(42.8)	262	(21.3)	42	(3.4)	678	(55.1)	346	(28.1)	207	(16.8)
No	23 544	490	(2.1)	1660	(7.1)	6427	(27.3)	10 260	(43.6)	4707	(9.9)	4353	(18.5)	5778	(24.5)	13 413	(56.9)
Lives alone																	
Yes	7386	189	(2.6)	685	(9.3)	2452	(33.2)	2941	(39.8)	1119	(15.2)	1787	(24.2)	2199	(29.8)	3400	(46.0)
No	16 878	386	(2.3)	1215	(7.2)	4245	(25.2)	7435	(44.1)	3597	(21.3)	2909	(17.2)	3801	(22.5)	10 168	(60.2)
Visual impairment																	
Yes	1711	77	(4.5)	226	(13.2)	717	(41.9)	571	(33.4)	120	(7.0)	729	(42.6)	604	(35.3)	378	(22.1)
No	23 064	513	(2.2)	1734	(7.5)	6237	(27.0)	9951	(43.2)	4629	(20.1)	4302	(18.7)	5520	(23.9)	13 242	(57.4)
Hearing impairment																	
Yes	4934	134	(2.7)	452	(9.2)	1805	(36.6)	1998	(40.5)	545	(11.0)	1415	(28.7)	1640	(33.2)	1879	(38.1)
No	19 841	456	(2.3)	1508	(7.6)	5149	(26.0)	8524	(43.0)	4204	(21.1)	3616	(18.2)	4484	(22.6)	11 741	(59.2)
Learning difficulty																	
Yes	307	19	(6.2)	49	(16.0)	113	(36.8)	101	(32.9)	25	(8.1)	153	(49.8)	79	(25.7)	75	(24.4)
No	24 468	571	(2.3)	1 911	(7.8)	6841	(28.0)	10 421	(42.6)	4724	(19.3)	4878	(19.9)	6045	(24.7)	13 545	(55.4)

### Associations of deprivation with self-reported health and day-to-day living

**Figure 2:**
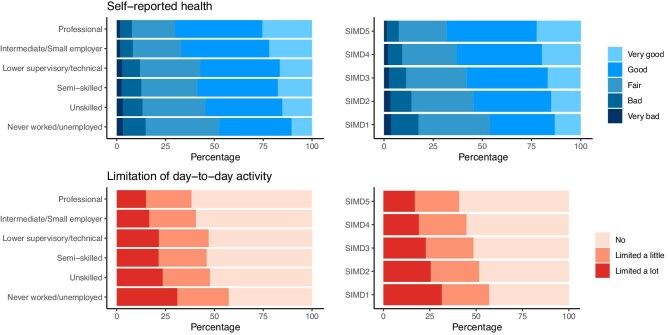
Age-standardized self-reported reported health and limitation of day-to-day activity among people presenting with kidney disease living in different household socioeconomic classifications (left), and neighbourhood socioeconomic quintiles (right) (SIMD5 = most affluent, SIMD1 = most deprived neighbourhood).

**Figure 3:**
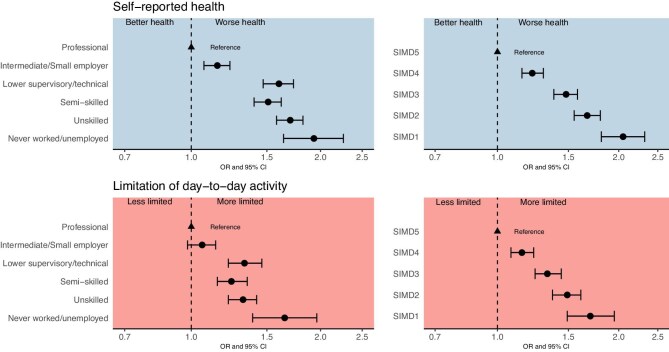
Independent associations between household (left) and neighbourhood (right) socioeconomic classifications and self-reported health (top row), and day-to-day activity limitation (bottom row).

Figure 2 illustrates differences in age-standardized self-reported health and day-to-day activity limitation by individual and neighbourhood socioeconomic classification. Across both outcomes and socioeconomic measures, greater deprivation was associated with worse health and more limitation in daily activities independent of age. As demonstrated in proportional odds models (Tables 3 and 4), after accounting for age, sex, and presenting year (model step A) those living in deprived circumstances were twice as likely to report experiencing worse health or being more limited in their ability to do daily activities. This changed little even after (model step B) adjusting for comorbidities and physical impairments, and (model step C) adjusting for mental health and living alone. As also illustrated in the final model in Fig. 3, compared with those living in affluent circumstances, people in the most deprived neighbourhood and households experienced greater odds of worse self-reported health (respective ORs 2.05, 1.81–2.32 SIMD 1 vs 5; 1.93, 1.64, 2.26 unemployed vs professionals), and of more limitation in day-to-day activities (respective ORs 1.70, 1.49–1.95 SIMD 1 vs 5; 1.65, 1.39–1.96 unemployed vs professionals).

**Table 3:** Association between socioeconomic deprivation and self-reported health.

	Step A age-sex adjusted		Step B^a^ + comorbidities and impairments		Step C^b^ + mental health and isolation	
Odds ratios for worse self-reported health	OR	95% CI	OR	95% CI	OR	95% CI
Neighbourhood classification						
(most affluent) SIMD 5	1.00	(ref)	1.00	(ref)	1.00	(ref)
SIMD 4	1.24	(1.17,1.32)	1.22	(1.15,1.29)	1.22	(1.15,1.30)
SIMD 3	1.53	(1.43, 1.63)	1.47	(1.38,1.57)	1.48	(1.38,1.58)
SIMD 2	1.86	(1.73,1.99)	1.70	(1.58,1.82)	1.67	(1.55,1.80)
(most deprived) SIMD 1	2.49	(1.21,2.81)	2.07	(1.83,2.34)	2.05	(1.81,2.32)
Household classification						
Professional	1.00	(ref)	1.00	(ref)	1.00	(ref)
Intermediate	1.18	(1.10,1.26)	1.15	(1.08,1.23)	1.15	(1.07,1.23)
Technical	1.72	(1.58,1.86)	1.59	(1.47,1.73)	1.60	(1.47,1.73)
Semi-skilled	1.65	(1.54,1.77)	1.54	(1.43,1.65)	1.51	(1.40,1.62)
Unskilled	1.91	(1.78,2.05)	1.72	(1.61,1.85)	1.70	(1.58,1.82)
Unemployed	2.45	(2.10,2.87)	2.01	(1.71,2.36)	1.93	(1.64,2.26)

Abbreviations: CI, confidence interval; SOR, odds ratio from a proportional odds model; ref, reference.

a Comorbidities included diabetes, chronic pulmonary disease, coronary heart disease, heart failure, hypertension, peripheral arterial disease, atrial fibrillation, stroke, and cancer. Impairments included hearing, vision, and learning difficulties.

b Based on mental health and living alone status as self-reported in the Census.

**Table 4:** Association between socioeconomic deprivation and limitation of day-to-day activities.

	Step A age-sex adjusted		Step B^a^+ comorbidities and impairments		Step C^b^+ mental health and isolation	
Odds ratios for more limitation in day-to-day activities	OR	95% CI	OR	95% CI	OR	95% CI
Neighbourhood classification						
(most affluent) SIMD 5	1.00	(ref)	1.00	(ref)	1.00	(ref)
SIMD 4	1.17	(1.1,1.25)	1.14	(1.06,1.21)	1.15	(1.08,1.23)
SIMD 3	1.39	(1.29, 1.49)	1.32	(1.23,1.42)	1.33	(1.24,1.44)
SIMD 2	1.64	(1.52,1.77)	1.48	(1.37,1.6)	1.49	(1.37,1.61)
(most deprived) SIMD 1	2.19	(1.94,2.49)	1.75	(1.54,1.99)	1.70	(1.49,1.95)
						
Household classification						
Professional	1.00	(ref)	1.00	(ref)	1.00	(ref)
Intermediate	1.1	(1.02,1.18)	1.07	(0.99,1.15)	1.06	(0.98,1.14)
Technical	1.45	(1.33,1.58)	1.32	(1.21,1.45)	1.33	(1.22,1.46)
Semi-skilled	1.37	(1.27,1.48)	1.27	(1.17,1.37)	1.24	(1.15,1.35)
Unskilled	1.5	(1.39,1.62)	1.33	(1.23,1.44)	1.32	(1.22,1.42)
Unemployed	2.29	(1.94,2.7)	1.78	(1.48,2.08)	1.65	(1.39,1.96)

Abbreviations: CI, confidence interval; OR, odds ratio from a proportional odds model; ref, reference.

a Comorbidities included diabetes, chronic pulmonary disease, coronary heart disease, heart failure, hypertension, peripheral arterial disease, atrial fibrillation, stroke, and cancer. Impairments included hearing, vision, and learning difficulties.

b Based on mental health and living alone status as self-reported in the Census.

### Associations stratified by sex, mental health, and age

**Figure 4:**
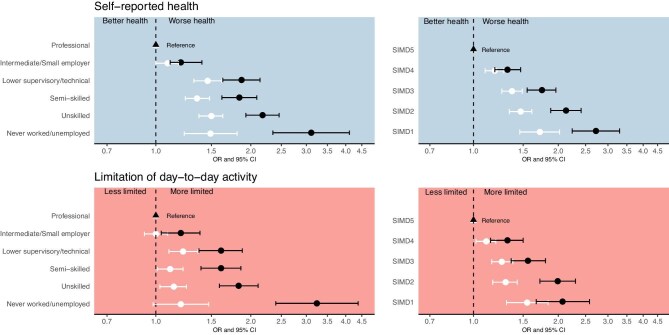
Independent associations between household (left) and neighbourhood (right) socioeconomic classifications and self-reported health (top row), day-to-day activity limitation (bottom) for people of working age (black) and older age (white).

Supplementary Tables 3–5 provide further information about the interaction sensitivity analyses. Across the 12 models there was limited borderline statistical evidence of an interaction for sex or mental health, directing us to stratified analyses. People with mental health conditions reported worse health and more activity limitation (see Table 2), but over and above this mental health did not substantially alter the associative patterns between deprivation and worse reported health (Supplementary Table 3). Females and males reported similar health and activity limitation overall (Table 1), and again there was no substantial difference in the associative patterns between deprivation and worse reported health (Supplementary Table 4). In contrast, all interactions tests were strongly significant for age (*P *< .0001). In stratified analysis, the relationship between deprivation and worse health and activity limitation was similar in pattern across ages, but substantially more pronounced among those of working age (18–65 years) such that those in deprived circumstances reported 3-fold greater odds of worse health and limitation of day-to-day activities (Fig. 4, Supplementary Table 5). This final model incorporating the age interaction is further illustrated in Supplementary Fig. 4, showing that a higher proportion of 50-year-old females living in a deprived neighbourhood would report worse self-reported health than 80-year-old females living in an affluent neighbourhood.

## DISCUSSION

In this large population study, irrespective of how deprivation was measured, at the point of newly presenting with kidney disease, people living in deprived communities were twice as likely to have reported poorer health and limitations in daily life activities. This pattern of inequity was evident after we accounted for age, sex, morbidities, physical impairments, mental health, and social isolation, and was even more pronounced among those of working age (3-fold). Moreover, the burden of health on daily life was commonly experienced: even at this early point in the clinical course almost of half of people reported that their health was affecting day-to-day life. Given the high priority placed on ‘life participation’ for people with CKD, this represents a significant, unmet, and inequitable need.

This analysis adds a lens of socioeconomic deprivation at a population level to complement existing studies that have described the overall health or symptom burden of people with kidney disease predominantly through selective recruitment of those under specialist/secondary care follow up [5–8, 43–50]. Further recent studies of the subset of people attending outpatient kidney clinics have identified potentially modifiable factors that affect quality of life of those with kidney disease including mental health, health literacy, smoking, obesity, polypharmacy, physical activity, and mental health [7, 45, 46, 51–58]. While associations between deprivation and self-reported health have been evaluated for cancer and cardiovascular disease, we were unable to find any previous studies for people with a new diagnosis or early-stage of kidney disease [24–27], but here report a large proportion of people limited by health with a large gap between affluent and deprived communities. In addition, this analysis also builds on the striking disparities in mortality outcomes for people with early kidney disease that we previously reported for this population in other work [14].

While the disparities are striking, the explanations and solutions are complex. Our analysis is cross-sectional and describes the daily limitations of people with a new presentation of kidney disease rather than due to kidney disease. Kidney disease itself is difficult to disentangle from co-existent long-term conditions that are commonplace. Further, while daily limitations were not commonly reported in the general population, we were unable to provide a non-kidney group in our analysis. We cannot attribute causality, but it is plausible that the limitations may represent a combined burden of multiple co-existing long-term conditions, perceived needs and available support to participate in daily life. Irrespective of cause, these are real-world experiences that are both inequitable and commonly experienced in the presence of long-term conditions such as kidney disease, which health professionals and services should be prepared to support across biopsychosocial dimensions of care. In the UK, the Long-Term Plan for the health service recognizes that long-term conditions including mental health, disabilities, and other conditions are a barrier to gaining and retaining employment, in addition to contributing to substantial societal loss of wellbeing and costs of £100 billion annually [59]. Our analysis provides a case that people of working age with kidney diseases are a large subset of those affected.

These findings should challenge how we organize our kidney services across primary, secondary, and social care to holistically address disadvantaged and under-served communities. If guidelines and pathways focus only on medical kidney and cardiovascular risk prevention, prescribing, and specialist referral criteria [60], there is a danger that important concerns may be left out of conversations or delegitimized [61]. It may be helpful to frame checks of kidney health as an opportunity to direct health professionals to identify, acknowledge and support the daily living concerns that many people living with kidney disease face, especially in contexts of socioeconomic disadvantage and living with multiple long-term conditions. This may benefit from a broader biopsychosocial approach supported by a wider primary care workforce and links to local community and neighbourhood resources [62, 63]. A recent qualitative evidence synthesis of the experiences of people living with multiple long-term conditions highlighted the impacts on daily life as multifaceted, cumulative and complex, comprising not only interactions with health services but many aspects such as emotions, finances, and a need to adapt to new and changing personal realities [64]. The review reported that these effects may not be apparent to healthcare professionals, and that current health systems and policies are poorly equipped to meet the needs.

Strengths of our analysis include a unique whole-population linkage of routine health and self-reported Census entries for all people presenting with kidney disease irrespective of their knowledge of their condition. This enabled us to assess the population-level implications of socioeconomic deprivation without creating biases that are inherent in processes of recruitment and consenting for surveys or interviews. For instance, a directed survey within a kidney clinic setting would represent a small (more severe) subset of people with kidney disease (sampling bias), with the impact of health on wellbeing framed around a known diagnosis (response bias). We also demonstrated consistency of relationships by using deprivation measures at both a neighbourhood and household level.

Common to all observational studies, our analysis could only capture the blood tests and Census entries as they were recorded, and therefore may have missed the difficulties faced by some people with unidentified kidney disease who may be hidden even within unselected population datasets. We have mitigated this by our use of an inclusive whole-population kidney definition, and a participatory approach to interpretation of findings, incorporating patient, public, clinical, and methodological stakeholders. Nevertheless, the inclusiveness of our criteria (new onset kidney impairment) means that our cohort should not be regarded as representing strictly confirmed cases of KDIGO CKD. Similarly, while we were able to account for the presence of other long-term conditions by adjusting for hospital episode diagnoses and patient self-reported conditions, we did not have access to conditions solely cared for within primary care, which may include musculoskeletal conditions or mental health conditions if people chose not to report them in the Census. We also note that a necessary trade-off of describing self-reported health at a population-wide level is some loss of granularity, such as quality of life dimensions of mobility, fatigue, and cognition. Even so, the two questions within the Census represent rigorously validated global assessment measures covering overall health and daily life that are central to societal decisions, reports, and policies of wellbeing and healthy life expectancy. Furthermore, our findings for people living in Scotland, may not be applicable to other countries, although we believe it is plausible to expect a similar pattern to exist in other European countries.

In summary, the association of deprivation and kidney disease with daily living and wellbeing represents an additional dimension of kidney health inequity that is substantial. Even at the earliest stages of newly presenting with kidney disease, people from deprived neighbourhoods and households are twice as likely to report worse general health and greater limitation in the activities of day-to-day life. These differences were even more pronounced for people of working age. These are pressing inequities that warrant attention to ensure that future models of kidney care more fairly support the challenges faced by those in greatest need.

## Supplementary Material

sfaf397_Supplemental_File

## Data Availability

Data access would require approval by Scotland’s Public Benefit and Privacy Panel for Health, Scotland’s Statistics Public Benefit and Privacy Panel, and appropriate ethical committees. Information on how researchers may make requests to obtain similar datasets from the health research dataset custodians may be provided upon request.
