# Supplementary material for: Deprivation and limitations in daily life in new onset kidney disease: a population study
Source: Clin Kidney J. 2025 Dec 17;19(1):sfaf397. doi: 10.1093/ckj/sfaf397 (PMC12828231; doi:10.1093/ckj/sfaf397)

Supplementary material for:

**Deprivation and limitations in daily life in new onset kidney disease: a population study**

Eilidh Cowan^1^ **(Lead author)**

Samira Bell MBChB, MD^3^

Prof Corri Black MB ChB, MSc^2^

Prof Tom Blakeman MBChB, PhD^4^

Prof Simon Fraser MBChB, PhD^5^

Audrey Hughes (patient partner)^6^

Buse Keskindag PhD^7^

Shona Methven MBChB, PhD^2^

Mintu Nath PhD^1^

Prof Dorothea Nitsch MSc Dr.med^8,9^

Magdalena Rzewuska Diaz PhD^7^

Nicole Scholes-Robertson PhD^10^

Simon Sawhney MBChB, PhD^1,2^ **(Corresponding author: simon.sawhney@abdn.ac.uk)**

^1^Aberdeen Centre for Health Data Science; University of Aberdeen

^2^NHS Grampian

^3^Division of Population Health and Genomics, School of Medicine; University of Dundee

^4^Centre for Primary Care, Institute of Population Health; University of Manchester

^5^School of Primary Care, Population Sciences and Medical Education; University of Southampton

^6^Grampian Kidney Patient Association

^7^Aberdeen Centre for Evaluation; University of Aberdeen

^8^UK Kidney Association; Bristol

^9^London School of Hygiene and Tropical Medicine

^10^Sydney School of Public Health; University of Sydney

Supplementary methods…………………………………………………………………………………………………………………page 3

Supplementary figure 1 – Description of cohort formation..……………………………………………………………page 4

Supplementary figure 2 – Conceptual diagram of relationships and approach to modelling……………page 5

Supplementary figure 3 – Limitation of day-today activities in the years leading up to new kidney disease presentation……………………..……………………………………………………………………………………………………………page 6

Supplementary table 1 – Categories of the Household National Statistics Socioeconomic Classification (NS-SEC) and example occupations…………………………………………………………………………………………………page 7

Supplementary table 2 – Additional characteristics by self-reported health status and limitation of day-to-day activity…………………………………………………………………………………………………………………………………page 8

Supplementary table 3 – Association between deprivation and self-reported health and limitation of day-to-day activities stratified by presence of a mental health condition………………………………………………page 9

Supplementary table 4 – Association between deprivation and self-reported health and limitation of day-to-day activities stratified by sex.………………………………………………………………………………………………….page 10

Supplementary table 5 – Association between deprivation and self-reported health and limitation of day-to-day activities stratified by working age...……………………………………………………………………………….…page 11

Supplementary figure 4 – Illustrative example based on predictions from the final models.…………page 12

**Supplementary methods**

*Ethical Statement*

This work was conducted with approvals from NHS Grampian Caldicott, Northwest Research Ethics Committee (19/NW/0552), NHS Research and Development, and the Statistics Public Benefit and Privacy Panel of the Scottish Government (1920-0075).

*Participatory analytic approach*

This analysis was part of the KINDER study (Kidney Inequalities: Needs Data Experiences Response), a Scottish Government-funded mixed-methods study involving a critical realist approach to explore how and why health inequities are experienced^1-3^. The study idea was initially conceived by our patient partners (led by co-author AH, chair of Grampian Kidney Patient Association). Each element of the statistical model design was developed with deliberations between qualitative and quantitative analysts, study leads (primary care, nephrology, and counselling practitioners), methods experts, clinical stakeholders, and public and patient partners. Results were interpreted using qualitative insights from patients and primary care professionals to understand social contexts and potential mechanisms.

References

1. Baum F, Smith D: Participatory action research. J Epidemiol Community Health 60(10):854–857, 2006

2. Eastwood J: Making a realist turn: Applying a critical realist translational social epidemiology methodology to the design and evaluation of complex integrated care interventions. Int J Integr Care 19(3):7, 2019

3. University of Aberdeen: KINDER: Understanding and reducing inequalities in kidney health care and outcomes in scotland, 2025, <https://www.abdn.ac.uk/ace/what-we-do/research/projects-a-z/kinder-understanding-and-reducing-inequalities-in-kidney-health-care-and-outcomes-in-scotland-356/>

Supplementary figure 1 – Description of cohort formation
Note: From 458,897 adult residents of Grampian who responded to the mandatory census, four parallel cohorts were created covering all incident cases of people crossing a threshold eGFR <60, <45, <30 ml/min/1.73m^2^ and acute kidney disease (AKD) as described in Sawhney et al, Kidney Int 106(5):928–942, 2024. We calculated eGFR using the Chronic Kidney Disease Epidemiology Consortium (CKD EPI 2009) without correction for race (Levey et al, Ann Intern Med 150(9):604–612, 2009) and used a Kidney Disease: Improving Global Outcomes (KDIGO) aligned algorithm to identify people with AKD (Sawhney et al, NDT 39(3):426-435, 2024). We applied a two-year exclusion period (2009-2010) to avoid a prevalent pool effect of mixing prevalent and incident patients. The cohort of incident eGFR <60 ml/min/1.73m^2^ was used in the main analysis and is summarised below.


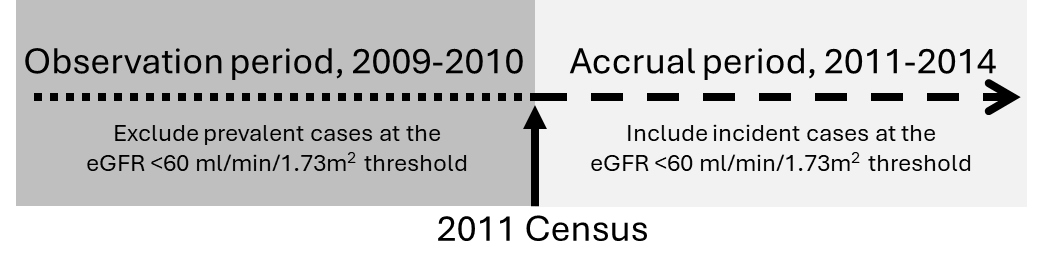


Supplementary figure 2 – Limitation of day-today activities in the years leading up to new kidney disease presentation
Note: We studied those with new evidence of reduced kidney function (eGFR<60 ml/min/1.73m2), 2011-2014 and their prior census responses. This was to avoid mis-attributing the burden of health on wellbeing experienced by prevalent rather than incident patients, which would have over-stated the burden at first presentation by including people with pre-existing longstanding kidney disease. As long-term health can worsen in the lead up to a new kidney presentation, based on *a priori* consultation with our patient and clinical experts, we excluded anyone presenting after 2014 (above the dotted line). Collectively, this ensured that we will not have over-reported burden of health on wellbeing in advance of kidney presentation, but may have conservatively under-estimated it. Additionally, presenting year was adjusted within the model to account for any residual imbalances between household deprivation categories.

Supplementary figure 3 – Conceptual diagram of relationships and approach to modelling
Note: Red text denotes additional variables added at each stage of model building. Stage C includes adjustment of potential mediators. We confirmed the inherent proportional odds assumption (i.e. that deprivation is associated with worse self-reported health both in increasing likelihood of health being “very bad” rather than “bad”, and “fair” rather than “good”), with a Brant-Wald test to check evidence of statistical departure from proportionality, as well as confirmatory checks of consistency of odds ratios across separate regression fits of each level of the multiple ordered levels of the outcome.


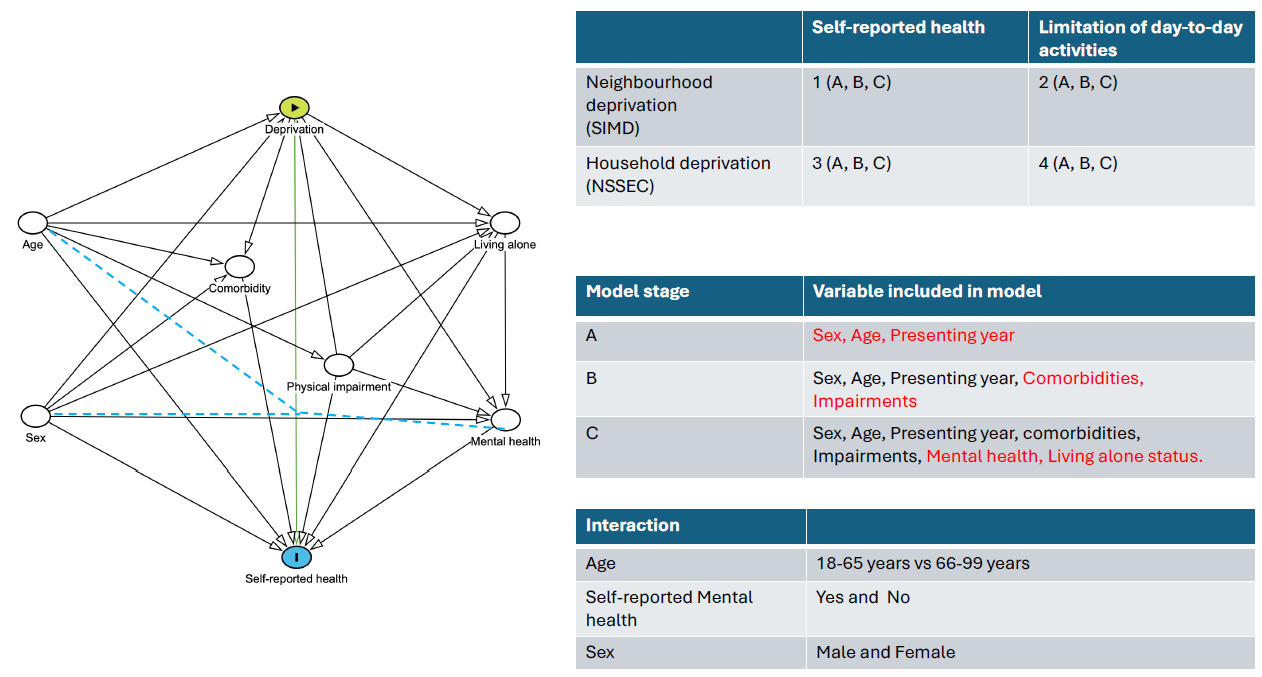


| **Category** | **NS-SEC definition** | **Occupation examples** |
| --- | --- | --- |
| 1 & 2 | Managers and professionals | Lawyers, architects, medical doctors, chief executives, economists, social workers, nurses, journalists, retail managers, teachers |
| 3 & 4 | Intermediate occupations and small employers | Paramedics, nursery nurses, farmers, shopkeepers, taxi drivers, driving instructors, window cleaners |
| 5 | Lower supervisory and technical occupations | Mechanics, chefs, train drivers, plumbers, electricians |
| 6 | Semi-routine (“semi-skilled” occupations) | Traffic wardens, receptionists, shelf-stackers, care workers, telephone salespersons |
| 7 | Routine (“unskilled” or casual work) | Bar staff, cleaners, labourers, lorry drivers |
| 8 | Never worked or long term unemployed | No regular employment |

Supplementary table 1 – Categories of the Household National Statistics Socioeconomic Classification (NS-SEC)
and example occupations (from Sawhney et al., Kidney Int 2024)

|  | **Total** | **Very bad** | | **Bad** | | **Fair** | | **Good** | | **Very good** | |  | **Limited a lot** | | **Limited a little** | | **Not limited** | |
| --- | --- | --- | --- | --- | --- | --- | --- | --- | --- | --- | --- | --- | --- | --- | --- | --- | --- | --- |
|  | **N** | **N** | **Row %** | **N** | **Row %** | **N** | **Row %** | **N** | **Row %** | **N** | **Row %** |  | **N** | **Row %** | **N** | **Row %** | **N** | **Row %** |
| **Total** | 24775 | 590 | (2.4) | 1960 | (8.0) | 6954 | (28.0) | 10522 | (42.5) | 4749 | (19.2) |  | 5031 | (20.3) | 6124 | (24.7) | 13620 | (55.0) |
| **Comorbidities at census** |  |  |  |  |  |  |  |  |  |  |  |  |  |  |  |  |  |  |
| Diabetes | 1467 | 89 | (6.1) | 268 | (18.3) | 585 | (39.9) | 465 | (31.7) | 60 | (4.1) |  | 543 | (37.0) | 424 | (28.9) | 500 | (34.1) |
| Chronic pulmonary disease | 1859 | 136 | (7.3) | 390 | (21.0) | 708 | (38.1) | 525 | (28.2) | 100 | (5.4) |  | 758 | (40.8) | 551 | (29.6) | 550 | (29.6) |
| Coronary heart disease | 1368 | 69 | (5.0) | 203 | (14.8) | 572 | (41.8) | 457 | (33.4) | 67 | (4.9) |  | 48- | (35.1) | 445 | (32.5) | 443 | (32.4) |
| Heart failure | 569 | 44 | (7.7) | 107 | (18.8) | 253 | (44.5) | 141 | (24.8) | 24 | (4.2) |  | 246 | (43.2) | 196 | (34.4) | 127 | (22.3) |
| Hypertension | 4663 | 181 | (3.9) | 582 | (12.5) | 1770 | (38.0) | 1742 | (37.4) | 388 | (8.3) |  | 1422 | (30.5) | 1440 | (30.9) | 1801 | (38.6) |
| Peripheral arterial disease | 618 | 47 | (7.6) | 123 | (19.9) | 262 | (42.4) | 158 | (25.6) | 28 | (4.5) |  | 292 | (47.2) | 189 | (30.6) | 137 | (22.2) |
| Atrial fibrillation | 1086 | 43 | (3.9) | 169 | (15.6) | 472 | (43.5) | 346 | (31.9) | 56 | (5.2) |  | 374 | (34.4) | 388 | (35.7) | 324 | (29.8) |
| Stroke | 724 | 44 | (6.1) | 140 | (19.3) | 325 | (44.9) | 185 | (25.6) | 30 | (4.1) |  | 353 | (48.8) | 205 | (28.3) | 166 | (22.9) |
| Cancer | 1493 | 83 | (5.5) | 190 | (12.7) | 539 | (36.1) | 542 | (36.3) | 139 | (9.3) |  | 409 | (27.4) | 432 | (28.9) | 652 | (43.7) |
| **Year of presentation** |  |  |  |  |  |  |  |  |  |  |  |  |  |  |  |  |  |  |
| 2011 | 5942 | 207 | (3.5) | 573 | (9.6) | 1927 | (32.4) | 2408 | (40.5) | 827 | (13.9) |  | 1567 | (26.4) | 1600 | (26.9) | 2775 | (46.7) |
| 2012 | 4883 | 121 | (2.5) | 429 | (8.8) | 1452 | (29.7) | 2002 | (41.0) | 879 | (18.0) |  | 1083 | (22.2) | 1271 | (26.0) | 2529 | (51.8) |
| 2013 | 4908 | 115 | (2.3) | 368 | (7.5) | 1297 | (26.4) | 2151 | (43.8) | 977 | (19.9) |  | 921 | (18.8) | 1201 | (24.5) | 2786 | (56.8) |
| 2014 | 4667 | 86 | (1.8) | 302 | (6.5) | 1201 | (25.7) | 2037 | (43.6) | 1041 | (22.3) |  | 782 | (16.8) | 1085 | (23.2) | 2800 | (60.0) |
| 2015 | 4375 | 61 | (1.4) | 288 | (6.6) | 1077 | (24.6) | 1924 | (44.0) | 1025 | (23.4) |  | 678 | (15.5) | 967 | (22.1) | 2730 | (62.4) |
|  |  |  |  |  |  |  |  |  |  |  |  |  |  |  |  |  |  |  |
| **Language difficulty** |  |  |  |  |  |  |  |  |  |  |  |  |  |  |  |  |  |  |
| Yes | 1771 | 69 | (3.9) | 177 | (10.0) | 587 | (33.1) | 700 | (39.5) | 238 | (13.4) |  | 543 | (31) | 423 | (24) | 805 | (46) |
| No | 23004 | 521 | (2.3) | 1783 | (7.8) | 6367 | (27.7) | 9822 | (42.7) | 4511 | (19.6) |  | 4488 | (20) | 5701 | (25) | 12815 | (56) |
|  |  |  |  |  |  |  |  |  |  |  |  |  |  |  |  |  |  |  |
| **Car access** |  |  |  |  |  |  |  |  |  |  |  |  |  |  |  |  |  |  |
| Yes | 17968 | 370 | (2.1) | 1183 | (6.6) | 4348 | (24.2) | 8089 | (45.0) | 3978 | (22.1) |  | 2831 | (15.8) | 4088 | (22.8) | 11049 | (61.5) |
| No | 6296 | 205 | (3.3) | 717 | (11.4) | 2349 | (37.3) | 2287 | (36.3) | 738 | (11.7) |  | 1865 | (29.6) | 1912 | (30.4) | 2519 | (40.0) |
| **Rurality** |  |  |  |  |  |  |  |  |  |  |  |  |  |  |  |  |  |  |
| Rural | 7198 | 140 | (1.9) | 462 | (6.4) | 1910 | (26.5) | 3100 | (43.1) | 1586 | (22.0) |  | 1311 | (18.2) | 1681 | (23.4) | 4206 | (58.4) |
| Urban | 17440 | 449 | (2.6) | 1482 | (8.5) | 5007 | (28.7) | 7367 | (42.2) | 3135 | (18.0) |  | 3693 | (21.2) | 4407 | (25.3) | 9340 | (53.6) |

Supplementary table 2 – Additional characteristics by self-reported health status and limitation of day-to-day activity

|  | **Worse self-reported health** | | | | **More limitation in day-to-day activities** | | | |
| --- | --- | --- | --- | --- | --- | --- | --- | --- |
|  | **Mental health condition** | | **No mental health condition** | | **Mental health condition** | | **No mental health condition** | |
|  | **OR** | **95% CI** | **OR** | **95% CI** | **OR** | **95% CI** | **OR** | **95%CI** |
| **Neighbourhood classification** |  |  |  |  |  |  |  |  |
| (most affluent) SIMD 5 | 1.00 | (ref) | 1.00 | (ref) | 1.00 | (ref) | 1.00 | (ref) |
| SIMD 4 | 1.16 | (0.85,1.59) | 1.23 | (1.15,1.30) | 1.19 | (0.85,1.66) | 1.15 | (1.08,1.24) |
| SIMD 3 | 1.89 | (1.37,2.61) | 1.46 | (1.36,1.56) | 2.05 | (1.44,2.92) | 1.31 | (1.21,1.41) |
| SIMD 2 | 1.51 | (1.08,2.11) | 1.68 | (1.55,1.81) | 1.79 | (1.24,2.58) | 1.47 | (1.35,1.59) |
| (most deprived) SIMD 1 | 1.63 | (1.03,2.58) | 2.09 | (1.84,2.38) | 1.47 | (0.9,2.4) | 1.72 | (1.5,1.98) |
|  |  |  |  |  |  |  |  |  |
| **Household classification** |  |  |  |  |  |  |  |  |
| Professional | 1.00 | (ref) | 1.00 | (ref) | 1.00 | (ref) | 1.00 | (ref) |
| Intermediate | 1.09 | (0.77,1.55) | 1.15 | (1.07,1.23) | 1.07 | (0.74,1,55) | 1.06 | (0.98,1.14) |
| Technical | 1.61 | (1.10,2.38) | 1.59 | (1.46,1.73) | 1.76 | (1.14,2.07) | 1.31 | (1.20,1.44) |
| Semi-skilled | 1.83 | (1.29,2.60) | 1.49 | (1.38,1.60) | 1.65 | (1.12,2.41) | 1.22 | (1.13,1.33) |
| Unskilled | 1.65 | (1.19,2.29) | 1.7 | (1.58,1.83) | 1.41 | (0.98, 2.01) | 1.31 | (1.21,1.42) |
| Unemployed | 1.39 | (0.80,2.41) | 1.98 | (1.68,2.35) | 1.81 | (1.01,3.22) | 1.64 | (1.37,1.96) |

Supplementary table 3 – Association between deprivation and self-reported health and limitation of day-to-day activities stratified by presence of a mental health condition

Abbreviations: CI, confidence interval; SIMD, Scottish index of multiple deprivation; OR, odds ratio from a proportional odds model; ref, reference

Adjusted model includes comorbidities (diabetes, chronic pulmonary disease, coronary heart disease, heart failure, hypertension, peripheral arterial disease, atrial fibrillation, stroke, cancer) based on ICD-10 codes; and impairments (hearing, vision, and learning difficulties), mental health and living alone status as self-reported in the Census

Respective p-values for interaction of mental health with self-reported health and SIMD, activity limitation and SIMD, self-reported health and NSSEC, activity limitation and NSSEC were 0.15, 0.04, 0.52, and 0.66.

|  | **Worse self-reported health** | | | | **More limitation in day-to-day activities** | | | |
| --- | --- | --- | --- | --- | --- | --- | --- | --- |
|  | **Male** | | **Female** | | **Male** | | **Female** | |
|  | **OR** | **95% CI** | **OR** | **95% CI** | **OR** | **95% CI** | **OR** | **95% CI** |
| **Neighbourhood classification** |  |  |  |  |  |  |  |  |
| (most affluent) SIMD 5 | 1.00 | (ref) | 1.00 | (ref) | 1.00 | (ref) | 1.00 | (ref) |
| SIMD 4 | 1.25 | (1.14,1.37) | 1.19 | (1.10,1.30) | 1.21 | (1.09,1.34) | 1.11 | (1.01,1.22) |
| SIMD 3 | 1.50 | (1.36,2.66) | 1.45 | (1.33,1.59) | 1.37 | (1.23,1.54) | 1.30 | (1.18,1.44) |
| SIMD 2 | 1.69 | (1.51,1.88) | 1.66 | (1.50,1.83) | 1.62 | (1.43,1.83) | 1.39 | (1.25,1.55) |
| (most deprived) SIMD 1 | 2.10 | (1.75,2.52) | 2.00 | (1.69,2.37) | 1.72 | (1.41,2.1) | 1.69 | (1.41,2.02) |
|  |  |  |  |  |  |  |  |  |
| **Household classification** |  |  |  |  |  |  |  |  |
| Professional | 1.00 | (ref) | 1.00 | (ref) | 1.00 | (ref) | 1.00 | (ref) |
| Intermediate | 1.24 | (1.12,1.36) | 1.08 | (0.99,1.18) | 1.13 | (1.01,1.26) | 1.01 | (0.92,1.12) |
| Technical | 1.54 | (1.38,1.73) | 1.66 | (1.48,1.87) | 1.31 | (1.16,1.49) | 1.36 | (1.20,1.54) |
| Semi-skilled | 1.54 | (1.38,1.73) | 1.47 | (1.34,1.62) | 1.29 | (1.14,1.46) | 1.21 | (1.09,1.34) |
| Unskilled | 1.63 | (1.47,1.81) | 1.75 | (1.59,1.93) | 1.32 | (1.18, 1.48) | 1.30 | (1.17,1.45) |
| Unemployed | 1.71 | (1.26,2.33) | 1.99 | (1.65,2.41) | 1.63 | (1.16,2.27) | 1.64 | (1.34,2.00) |

Supplementary table 4 – Association between deprivation and self-reported health and limitation of day-to-day activities stratified by sex

Abbreviations: CI, confidence interval; SIMD, Scottish index of multiple deprivation; OR, odds ratio from a proportional odds model; ref, reference

Adjusted model includes comorbidities (diabetes, chronic pulmonary disease, coronary heart disease, heart failure, hypertension, peripheral arterial disease, atrial fibrillation, stroke, cancer) based on ICD-10 codes; and impairments (hearing, vision, and learning difficulties), mental health and living alone status as self-reported in the Census

Respective p-values for interaction of sex with self-reported health and SIMD, activity limitation and SIMD, self-reported health and NSSEC, activity limitation and NSSEC were 0.96, 0.44, 0.06, and 0.75.

|  | **Worse self-reported health** | | | | **More limitation in day-to-day activities** | | | |
| --- | --- | --- | --- | --- | --- | --- | --- | --- |
|  | **Working age**  **18-65 years** | | **Non-working age**  **66-99 years** | | **Working age**  **18-65 years** | | **Non-working age**  **66-99 years** | |
|  | **OR** | **95% CI** | **OR** | **95% CI** | **OR** | **95% CI** | **OR** | **95% CI** |
| **Neighbourhood classification** |  |  |  |  |  |  |  |  |
| (most affluent) SIMD 5 | 1.00 | (ref) | 1.00 | (ref) | 1.00 | (ref) | 1.00 | (ref) |
| SIMD 4 | 1.32 | (1.19,1.47) | 1.19 | (1.10,1.28) | 1.32 | (1.15,1.50) | 1.11 | (1.02,1.20) |
| SIMD 3 | 1.75 | (1.55,1.96) | 1.37 | (1.26,1.49) | 1.56 | (1.36,1.80) | 1.26 | (1.16,1.38) |
| SIMD 2 | 2.13 | (1.88,2.41) | 1.47 | (1.34,1.61) | 1.99 | (1.72,2.30) | 1.30 | (1.17,1.43) |
| (most deprived) SIMD 1 | 2.72 | (2.24,3.30) | 1.72 | (1.46,2.02) | 2.07 | (1.67,2.58) | 1.55 | (1.31,1.84) |
|  |  |  |  |  |  |  |  |  |
| **Household classification** |  |  |  |  |  |  |  |  |
| Professional | 1.00 | (ref) | 1.00 | (ref) | 1.00 | (ref) | 1.00 | (ref) |
| Intermediate | 1.25 | (1.11,1.40) | 1.09 | (1.00,1.18) | 1.20 | (1.04,1.38) | 1.00 | (0.92,1.09) |
| Technical | 1.87 | (1.63,2.14) | 1.46 | (1.32,1.62) | 1.61 | (1.37,1.88) | 1.22 | (1.10,1.36) |
| Semi-skilled | 1.84 | (1.62,2.09) | 1.35 | (1.24,1.48) | 1.61 | (1.39,1.86) | 1.11 | (1.01,1.22) |
| Unskilled | 2.18 | (1.93,2.46) | 1.50 | (1.37,1.63) | 1.83 | (1.59, 2.11) | 1.14 | (1.03,1.25) |
| Unemployed | 3.11 | (2.35,4.11) | 1.49 | (1.23,1.81) | 3.24 | (2.40,4.38) | 1.20 | (0.98,1.47) |

Supplementary table 5 – Association between deprivation and self-reported health and limitation of day-to-day activities stratified by working age

Abbreviations: CI, confidence interval; SIMD, Scottish index of multiple deprivation; OR, odds ratio from a proportional odds model; ref, reference

Adjusted model includes comorbidities (diabetes, chronic pulmonary disease, coronary heart disease, heart failure, hypertension, peripheral arterial disease, atrial fibrillation, stroke, cancer) based on ICD-10 codes; and impairments (hearing, vision, and learning difficulties), mental health and living alone status as self-reported in the Census

Respective p-values for interaction of sex with self-reported health and SIMD, activity limitation and SIMD, self-reported health and NSSEC, activity limitation and NSSEC were <0.0001, <0.0001, <0.0001, and <0.0001.

Supplementary figure 4 – Illustrative example for 100 people based on the final models for (A) self-reported health, and (B) limitation of day-to-day activities
Notes: Model predictions for 100 female people newly presenting with eGFR <60 ml/min/1.73m^2^ who have no co-morbidities, no physical impairments, no self-reported mental health condition and are not living alone. Estimates provided according to neighbourhood deprivation quintile (most deprived vs most affluent neighbourhood), and age (50 years vs 80 years) and include the significant interaction term of age on the relationship between deprivation and self-reported health.

(3A) Based on the final model for self-reported health, among 100 people a higher proportion of 50 year old females living in a deprived area would report worse health (top left) than 80 year old females living in an affluent area (bottom right).

(3B) Based on the final model for limitation of day-to-day activities, among 100 people a similar proportion of 50 year old females living in a deprived area would report limitation in day-to-day activities to 80 year old females living in an affluent area.


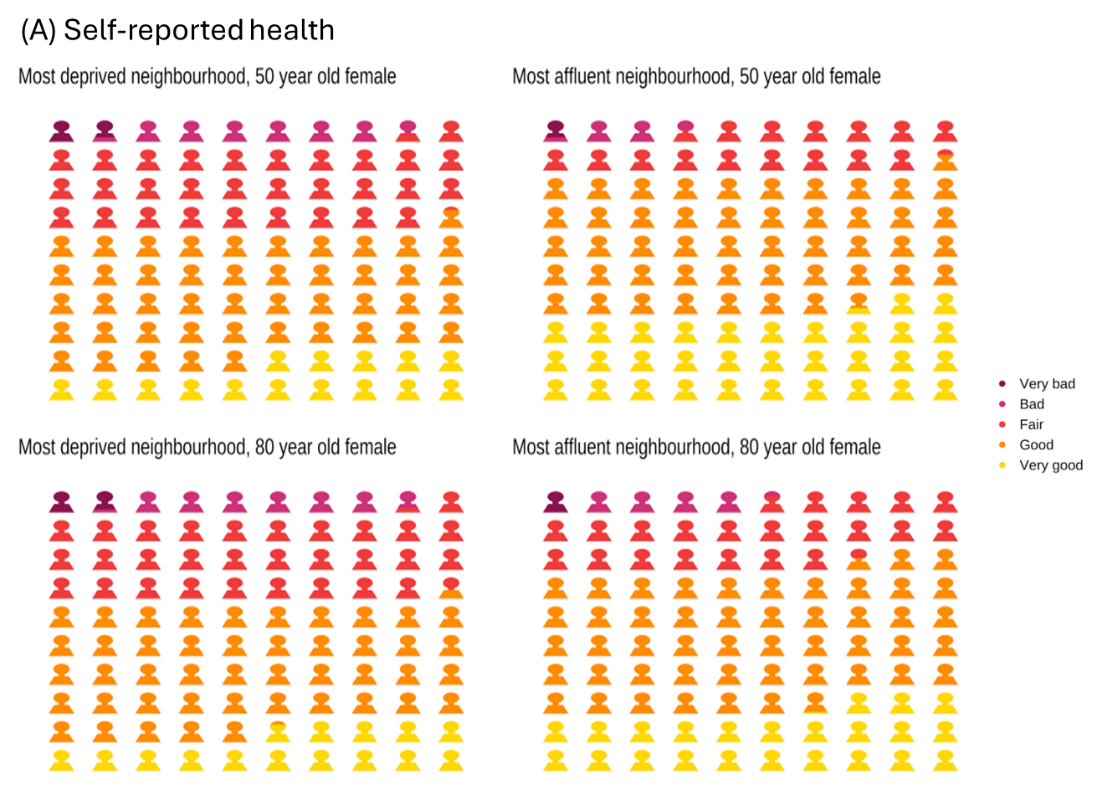


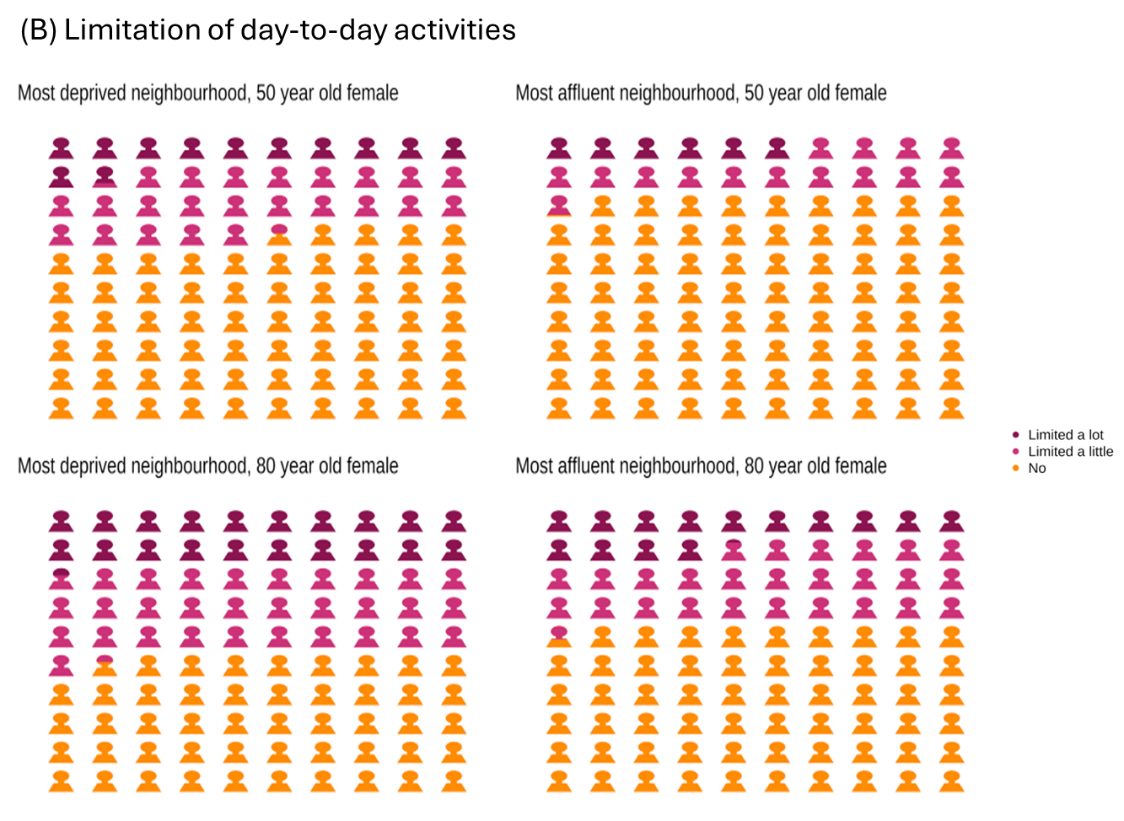

Supplement: sfaf397_Supplemental_File [file sfaf397_supplemental_file.docx]
